# Liquid Biopsy in Squamous Cell Carcinoma of the Esophagus and of the Head and Neck

**DOI:** 10.3389/fmed.2022.827297

**Published:** 2022-04-29

**Authors:** Razvan Iacob, Matei Mandea, Speranta Iacob, Catalina Pietrosanu, Doru Paul, Razvan Hainarosie, Cristian Gheorghe

**Affiliations:** ^1^University of Medicine and Pharmacy “Carol Davila”, Bucharest, Romania; ^2^Digestive Diseases and Liver Transplantation Center, Fundeni Clinical Institute, Bucharest, Romania; ^3^Center of Excellence in Translational Medicine, Fundeni Clinical Institute, Bucharest, Romania; ^4^Professor Doctor Dorin Hociota Institute of Phonoaudiology and Functional ENT Surgery, Bucharest, Romania; ^5^Division of Hematology and Medical Oncology, Department of Medicine, Weill Cornell Medicine, Cornell University, New York, NY, United States

**Keywords:** liquid biopsy, esophageal squamous cell carcinoma, head and neck squamous cell carcinoma, cell free DNA, circulating tumor cells, exosomes

## Abstract

Squamous cell carcinomas of the esophagus (ESCC) and of the head and neck (HNSCC) are two neoplasms that share common risk factors and have the same embryological origin, but a very different prognosis, the 5-year survival of HNSCC being almost double (40–50%) compared to the 5-year survival of ESCC (20%). Current guidelines emphasize the importance of screening for ESCC in patients diagnosed with head and neck cancers. A liquid biopsy is a novel tool for diagnosis, prognostic stratification, and personalized therapy. Liquid biopsy biomarkers for these two malignancies could help both their early detection, facilitate residual disease identification, and provide prognosis information. The present systematic review of the literature was aimed at describing the liquid biopsy biomarkers present in these two malignancies, with an emphasis on potential clinical applications.

## Introduction

Squamous cell carcinoma of the esophagus (ESCC) and of the head and neck are common neoplastic pathologies being responsible of over 500,000 deaths per year, with an increased incidence in the past few years. The head and neck squamous cell carcinoma (HNSCC) can arise from tissues of the oral cavity, oropharynx, hypopharynx, larynx, and nasopharynx. According to the GLOBOCAN 2020, the incidence of HNSCC has increased worldwide in the past decade, with more than 800,000 new cases every year, with cancer of the oral cavity and lips being the most frequent (1). The most important risk factors are smoking, human papillomavirus (HPV) infection, and alcohol abuse, with an increased number of cases in males (2–4). The diagnosis is often made in a late stage due to suboptimal conventional biomarkers for early-stage disease. 40% of patients are diagnosed when lymph nodes metastasis is present and the outcome is poor (2, 3). Currently, the diagnosis consists of imaging methods associated with tissue biopsy (3). Despite recent advances in locoregional therapies, up to 60% of HNSCC tumors will have a locoregional recurrence and additional 20% will develop distant metastasis, leading to treatment failure (5). The tumor HPV assessed by p16 expression is currently the most widely used biomarker in HNSCC (6). Overall, HNSCC has a 40–50% 5-year survival.

The ESCC is often diagnosed in a late stage, with a <20% survival at 5 years from diagnosis. The incidence has decreased in the past decade due to smoking cessation, but there are still over 600,000 new cases diagnosed every year (1). ESCC ranks as the sixth cause of cancer-related mortality worldwide, with an age-standardized rate/100,000 (ASR) of 5.6. Currently, screening for ESCC is recommended also for patients with a personal or familial history of head and neck cancers and is based on upper gastrointestinal endoscopy. The use of carbohydrate antigen 19-9 (CA 19-9) and carcinoembryonic antigen (CEA) do not have good sensitivity in detecting early cases of ESCC and are not recommended as routine screening tests in clinical practice (7–9).

Although HNSCC and ESCC share common risk factors, such as tobacco usage, alcohol abuse, male sex, and other risk factors are specific for each type, such as HPV infection and poor oral hygiene for HNSCC and hot food and beverages and changes in the oral microbiome for ESCC, respectively (4, 6, 8, 10).

Several studies have documented that the presence of HNSCC and ESCC in the same patient leads to decreased longevity and increased morbidity, especially when surgical resection is conducted for both the malignancies (11, 12). Currently, high-resolution chromoendoscopy or virtual chromoendoscopy using NBI or I-SCAN techniques is the gold standard for early diagnosis of ESCC and endoscopic resection of early ESCC is the best therapeutic option (13). As radiation therapy conducted in HNSCC might induce radiation esophagitis with or without strictures, alternative methods of molecular diagnosis that could indicate the potential coexistence of ESCC are clearly useful. Therefore, the identification of markers for early diagnosis and posttreatment disease monitoring are of paramount importance for both the malignancies, as early diagnosis is the cornerstone of curative therapies. Considering that the two anatomical sites share a common embryological origin and common risk factors, one could expect to find common molecular markers that could be used for the early detection and prognosis of both the malignancies. At the same time, tumor-specific molecular markers would further refine our ability to stratify patients both for the diagnostic and prognostic purposes and for treatment that is different in these two conditions.

## Liquid Biopsy as a Novel Tool for Diagnosis and Prognosis

Liquid biopsy has recently emerged as a new non-invasive technique of detecting blood circulating biomarkers. There are multiple applications of liquid biopsy currently investigated both for ESCC and HNSCC (3, 14, 15), for example, detection of circulating tumor cells (CTCs), circulating tumor DNA (ctDNA), and exosomes-based biomarkers (16, 17).

### Circulating Tumor Cells

Circulating tumor cells represent cancer cells derived from the primary site of neoplasia that are shed in the blood stream or in the lymphatics and are responsible for metastatic progression of the disease (16, 17). They can be used as diagnostic or prognosis markers, being useful for estimating the tumor dynamics. The detection of CTCs is based on markers expression on the surface and in the cytoplasm of the CTCs (17).

### Cell-Free DNA (CfDNA)

Cell-free DNA is released in the bloodstream during necrosis and apoptosis of cells. The tumoral source of cfDNA is represented by ctDNA and it is present along cfDNA originating from normal cells, as a fraction of the total circulating free DNA. Valuable tumor information like somatic mutations or methylation profiles could be detected through specific methods such as next-generation sequencing (NGS) and are important for early disease detection, detection of residual disease following therapy, personalized therapeutic tailoring, and prognostic estimation (15, 16, 18).

### Exosomes

Exosomes are stable extracellular vesicles of small size (40–150 nm), released by cells in various body fluids, containing proteins, microRNAs (miRNA), messenger RNA (mRNA), DNA, and long noncoding RNA (lncRNA) (16). miRNAs and lncRNAs are included in the category of regulatory noncoding RNAs. miRNA consists of 20–24 nucleotides, with important roles in cell differentiation, proliferation, apoptosis as well as in tumor suppression or promotion, by regulating the expression of multiple target genes (16, 19, 20). LncRNAs are RNAs consisting of over 200 nucleotides that do not code proteins and are released by tumoral cells also via exosomes (20).

## Systematic Literature Review

The scope of the present review was to systematically perform a literature search to identify biomarkers of liquid biopsy significant for diagnosis and prognosis of ESCC and HNSCC, respectively, with a special focus on biomarkers shared by the two cancers. Pubmed and CrossRef were used to search for original articles that were published between 2001 and 2021 using HNSCC and ESCC medical subject heading (MeSH) terms combined with terms liquid biopsy, CTCs, cfDNA, circulating-tumoral DNA, exosomes, and miRNA. The systematic literature search was conducted in March to August 2021. The literature was initially screened for types of liquid biopsy used in both the HNSCC and ESCC, further investigating whether markers are common or different depending on the type of cancer investigated.

## Circulating Tumor Cells in HNSCC

Two excellent recent publications, a systematic review and a meta-analysis have addressed the utility of CTCs in diagnostic and prognosis of HNSCC (17, 21). Most representative papers on this subject consist of nine prospective cohort studies and one retrospective cohort study (5, 22–30). The studies are heterogeneous in terms of disease staging, including either patients diagnosed in stages I–IV or only advanced disease cases (stages III–IV). Similarly, significant heterogeneity was noted about the timing of the study, before, during, or after treatment, or about the types of treatment (surgery, radiotherapy, or chemotherapy).

Several techniques were used for CTC isolation, such as negative enrichment immunofluorescence *in-situ* hybridization (Ne imFISH), the use of CellSearch or Clearcell FX platforms, by staining with immunofluorescent antibodies to specific cytokeratins, or by detection of Epidermal Growth Factor Receptor (EGFR) transcripts (23, 25, 26, 29, 31). The most frequently used definition of positive CTCs in HNSCC was by positivity for epithelial cell adhesion molecule (*EpCAM*) that is absent in normal blood mononuclear cells and negative CD45 staining that is specific to blood mononuclear cells (24, 32). The CellSearch system (Menarini Silicon Biosystems, Bologna, Italy) is, to date, the single Food and Drug Administration (FDA)-approved CTC detection and quantification platform. It performs immunomagnetic enrichment using ferrofluids with *EpCAM* antibodies coupled to an analyzer that captures images of isolated cells after staining with specific fluorescent antibodies. After immunomagnetic enrichment, recovered cells are permeabilized and stained with a nuclear stain (*DAPI*), and a fluorescent antibody conjugate against *CD45* (leukocyte marker) and *cytokeratins 8, 18*, and *19* (epithelial markers). To be considered a CTC, a cell must be *EpCAM* positive, stain positive for nuclear *DAPI*, have a positive cytoplasmic appearance for cytokeratins and a negative leucocytic *CD45* staining, having a diameter larger than 5 μm. The detection of more than 3–5 positive cells/7.5 ml of blood defines a positive sample and the detection limit is of 1 CTC/7.5 ml Ethylenediaminetetraacetic acid (EDTA)-blood (22, 31–34). The use of leucocytic marker *CD45* for negative selection combined with immunofluorescence staining methods that use antibodies against N-cadherin, *CD133*, and multi-cytokeratin was also employed for the detection of CTCs in HNSCC (30).

We have shown in Table 1 the most significant studies published on CTCs in HNSCC and ESCC, using the CellSearch platform. In both the malignancies, the level of CTCs detected correlated with poor prognosis, shorter disease-free survival, or reduced response to treatment, the data being more robust for ESCC (35, 37, 39, 41).

**Table 1:** Studies on circulating tumor cells in head and neck squamous cell carcinoma (HNSCC) and esophagus squamous cell carcinoma (ESCC) using CellSearch platform.

**Authors**	**Year of study**	**Tumor site**	**Stage of disease**	**Timepoint of analysis**	**Patients included**	**Utility**
Harris et al. (23)	2020	Oropharynx	III, IV	After CRT	47	Prognosis
Strati et al. (28)	2017	Oral cavity, Oropharynx, Hypopharynx, Larynx	I-IV	Before and after treatment	113	Prognosis
Grobe et al. (35)	2013	Oral cavity	I-IV	After surgery	80	Prognosis
Bozec et al. (36)	2013	Oral cavity, Oropharynx	III-IV	After treatment	49	Prognosis
Buglione et al. (22)	2012	Oropharynx, Nasopharynx, Larynx, Hypopharynx, Oral cavity, Paranasal sinuses	I-IV	Before treatment	73	Prognosis
Konczalla et al. (37)	2019	ESCC and AC	I-IV	Before surgery	23 ESCC, 53 AC	Prognosis
Li et al. (38)	2015	ESCC	I-IV	After treatment	61	Prognosis
Reeh et al. (39)	2015	ESCC and AC	I-IV	Before surgery	29 ESCC, 48 AC	Prognosis
Matsushita et al. (40)	2015	ESCC	II-IV	After treatment	90	Prognosis
Tanaka et al. (41)	2014	ESCC and AC	II-IV	Before CRT	34 ESCC, 3 AC	Prognosis

In several studies, expression of the programmed death ligand 1 (*PD-L1*) immunotherapy target in CTCs was investigated, in order to identify possible responders to pembrolizumab therapy and prognostic estimation (26, 28). A study from Strati et al. has quantified *PD-L1* expression on CTCs in peripheral blood samples of patients with HNSCC, using direct imaging through the CellSearch system, showing that the positivity to *PD-L1* is associated with poor prognosis (28).

The microfluidic platform system (CTC-Chip) and the isolation by size of epithelial tumor cells filtration methods (Isolation by Size of Tumor cells - ISET) use the size of the CTC specific to each tumor type. The use of dimension detecting techniques can help identify both the circulating tumor micro-emboli and clusters, which may express the *CD45* marker in some of the cells, therefore escaping methods that use *CD45* antibodies negative selection (21, 28).

The utility of detecting CTCs was, in most of the studies, for prognostic purpose or personalized therapy, with no current proven benefit for early diagnosis of HNSCC. The most important roles of detecting CTCs are for identifying future targets of treatment, to evaluate treatment response and disease recurrence (21, 32, 34). Further translational applications consist in establishing *in vitro* disease models based on circulating cancer stem cells that could be used for drug development and personalized therapy. Proposed head and neck cancer stem cell transcription factors and biomarkers are *CD44, CD133, ALDH, cMET, Oct-4, Nanog, Sox2* (42, 43). The identification of CTC subpopulations in patients with recurrent HNSCC may have future applications in developing novel antitumoral targeted therapies.

## Circulating Tumor Cells in ESCC

The current approach to therapy in ESCC is dependent on accurate estimation of the tumor staging and is conducted in a multidisciplinary way, involving endoscopist, radiologist, oncologist, and surgeon. An accurate pretherapeutic staging providing a clear picture of the disease spread is critical for optimal therapeutic choice, between endoscopic resection, surgery, radiochemotherapy, or systemic chemotherapy. As extraesophageal tumor recurrence is found in only approximately 50% of cases, one could imagine that micrometastases are frequently missed by current imaging modalities and responsible for frequent distant disease relapse (44). The detection of CTCs can improve our prognostic ability and enhance staging systems in ESCC. Our systematic literature search has identified several prospective cohort studies, published between 2007 and 2019 about the clinical utility of CTCs in ESCC. The studies included patients diagnosed in all stages of the disease, between I and IV as well as at different time points during the disease course, before or after treatment, consisting in surgery, radiotherapy, or chemotherapy (45, 46).

For detection and isolation of CTCs, there were used techniques selecting CTCs based on their antigenic surface profile and immune magnetic procedures or methods that use physical properties of the cells, such as cell size, specific density (gradient centrifugation enrichment), cellular electric charge properties (dielectrophoretic procedures), and the deformation ability (chip-based microfluidic CTC enrichment methods). Dedicated CTC isolation platforms such as CellSearch system and CanPatrol have also been used (40, 46–48). Usually, for detection of CTCs similar markers were used as in HNSCC, consisting in *CD45* negativity, and positivity for *DAPI, CEP8*, and cytokeratins (*CK18, CK19*, and *CK8*) and *EpCAM* (38, 40, 45, 46). The use of CanPatrol system by Chen et al. further classified CTCs in epithelial, mesenchymal, and hybrid, using expression of markers such as *EpCAM, CK, vimentin*, and *Twist* by fluorescent probes. The authors found that mesenchymal CTC count is directly correlated with clinical stage and response to chemotherapy (47).

Several studies have investigated the expression of survivin in CTCs. Survivin is a protein in the family of the inhibitors of apoptosis and is frequently expressed in numerous carcinomas and only rarely within normal cells. It can be detected in many types of samples such as blood, urine, and sputum. Survivin expressing CTCs are associated with invasiveness, high nodal status, and shorter overall survival (48, 49), however, no study concerning survivin expression in HNSCC was identified, so far. Similarly to HNSCC, the majority of the studies indicated that higher detection of CTCs count directly correlates with metastasis, recurrence, shorter disease-free survival, worse response to chemoradiotherapy (CRT), regardless of the expressing marker used for CTC detection (18, 38, 40, 46, 48–50).

The presence of CTCs in the blood is found in both the HNSCC and ESCC and, according to current knowledge, CTCs detection cannot discriminate between the two conditions.

## Cell-Free DNA and CTDNA in HNSCC

Cell-free DNA from the tumoral origin is called ctDNA and is represented by 140–170 bp DNA fragments that originate from necrotic or apoptotic tumoral cells. It can be used to detect tumor-specific genomic alterations such as somatic mutations and methylation profiles. It has been shown that the detection of specific genomic alterations of driver oncogenes, such as *TP53, EGFR*, and *KRAS* in ctDNA, has clinical utility for cancer screening, early diagnosis, and targeted treatment approach, identifying in patients with lung cancer, colorectal cancer, or pancreatic cancer subgroups that would benefit of specific chemotherapy regimens. According to the Catalogue of Somatic Mutations in Cancer (COSMIC) database, the most frequently mutated genes in HNSCC are *TP53* (44%), *LRP1B* (21%), *NOTCH1* (20%), *FAT1* (19%), and *KMT2D* (16%). More than 550 somatic variants in *TP53* have been described so far in HNSCC, the most frequently reported being *R175H, R248Q, R248W*, and *R273H* (51). One of the most important prognostic roles of identifying genomic alterations in cfDNA resides in the prediction of minimal residual disease, which is known as microscopic tumoral cells that are still present after a potentially curative treatment and that are associated with disease recurrence (52, 53).

Several PCR and NGS sequencing methods could be used to detect somatic mutations in cfDNA, such as digital droplet PCR (DDPCR), Competitive allele-specific TaqMan PCR (CAST-PCR), and NGS techniques with novel modifications and adaptations, such as safe-sequencing systems (Safe-SeqS) or Plasma sequencing (Plasma-Seq) (54–56). The various techniques have different limits of detection and could be used to investigate specific genomic alteration or a panel of target sequences. As shown in Table 2, the NGS and specified PCR techniques have been recently used to screen for and validate hotspot mutations in a targeted panel of genes, in HNSCC, by several study groups, including Braig et al. (61) and Wang et al. (63). Wang et al. have investigated genomic alterations in *TP53, CDKN2A, HRAS, NRAS, PIK3CA*, and HPV16 DNA in plasma or saliva from 93 patients with HNSCC, documenting a potential role for diagnosis of invasive HNSCC for this gene panel (63). Braig et al. identified by NGS that mutations in the RAS family genes are found in a subgroup of patients with HNSCC and correlate with disease progression, also after cetuximab treatment (61).

**Table 2:** Significant studies on cell-free DNA in HNSCC—clinical applications.

**Authors**	**Year**	**Type of alteration**	**Type of cancer**	**Genes**	**Stage of disease**	**Assay type**	**Timing of sampling**	**Utility**
Schmidt et al. (57)	2018	Mutation	HNSCC	*PIK3CA*	III-IV	Allele-specific Plex-PCR	Before treatment	Diagnosis and monitoring
Vos et al. (58)	2017	Methylation	HNSCC	*SEPT9, SHOX2*	-	ACTB triplex qPCR	Before treatment	Diagnosis
Perdomo et al. (59)	2017	Mutation	HNSCC	*TP53; NOTCH1, CDKN2A, CASP8, PTEN*	I-IV	NGS	-	Diagnosis
Schrock et al. (60)	2017	Methylation	HNSCC	*SEPT9, SHOX2*	I-IV	ACTB triplex qPCR	Before and after treatment	Diagnosis and prognosis
Braig et al. (61)	2016	Mutation	HNSCC	*EGFR exon 12; KRAS exon 2,3,4; NRAS exon 2,3,4; HRAS exon 2,3*	III-IV	NGS	After chemotherapy	Prognosis and treatment response
Mazurek et al. (62)	2015	Mutation	HNSCC	*KRAS G12C; p.E746-A750del EGFR*	I-IV	TaqMan genotyping	After CRT	Diagnosis
Wang et al. (63)	2015	Mutation	HNSCC	*TP53, PIK3CA, CDKN2A, FBXW7, HRAS, NRAS*	I-IV	Safe-SeqS PCR	Before treatment	Diagnosis
Mydlarz et al. (64)	2014	Methylation	HNSCC	*EDNRB, p16, DCC*	I-IV	Quantitative methylation specific PCR	Before treatment	Diagnosis
Yang et al. (65)	2014	Methylation	NPSCC	*RASSF1A, WIF1, DAPK1, RARB2*	I-IV	MS HRM PCR	Before treatment	Diagnosis and prognosis
Tian et al. (66)	2013	Methylation	NPSCC	*RASSF1, CDKN2A, DLEC1, DAPK1, UCHL1*	I-IV	qPCR	-	Diagnosis

Mazurek et al. and Perdomo et al. have shown that somatic alterations of either *KRAS* and *EGFR* or *TP53, NOTCH1, CDKN2A, CAP8*, and *PTEN* could be used for the diagnostic purpose by liquid biopsy in HNSCC (59, 62). Perdomo et al. have proposed that *TP53* genomic alterations could be a suitable biomarker for early detection of HNSCC in patients with HPV negative (59).

Aberrant methylation profiles are genomic alteration marking the early events of carcinogenesis and quantitative measure of methylation in specific gene loci has emerged as a novel tool for early diagnosis, prognosis, and disease monitoring (60). Methylation can be determined using different technologies, such as quantitative PCR (qPCR) and methylation sensitive high-resolution melting PCR (MS-HRM PCR) (53, 60). In HNSCC, the most frequently investigated methylated targets were *SEPT9, SHOX2, DAPK1, RASSF1A*, and *CDKN2A* genes (53, 58, 60). Gene methylations of *SEPT9* and *SHOX2* were identified in patients with different types of HNSCC, regardless of tumor stage, and hypermethylation could be used as a valuable biomarker for diagnosis and prognosis. For diagnostic purpose, studies have shown a good specificity, over 90%, but a rather low sensitivity (about 50%) (58, 60). By investigating methylation profiles in cfDNA in patients with nasopharyngeal-squamous cell carcinoma, Tian et al. and Yang et al. have proposed that investigating a panel of markers leads to better results that a single methylation target (65, 66). Methylation of *CDKN2A* had a sensitivity of 22.5% and a specificity of 97%, methylation of *DAPK1*, a sensitivity of 51.4%, and a specificity of 90%, whereas the panel consisting of *CDKN2A, DLEC1, DAPK1*, and *UCHL1* was found to have the highest specificity and sensitivity for HNSCC detection (66). In another study, the use of a methylation panel consisting of *RASSF1, WIF1, DAPK1*, and *RARB2* had a sensitivity of 95.8% and a detection rate of early-stage disease of 90%, showing great promise for clinical application. The highest specificity was identified for methylations of *RASSF1A* gene (65).

## Cell-Free DNA and CtDNA in ESCC

Tumor-specific gene alterations detection in ESCC are currently explored as a novel type of biomarker useful for diagnosis and prognosis. In the COSMIC database, there are to date 2,236 samples of ESCC genomic profiles reported, indicating that the most frequently mutated genes are *TP53* (58%), *KMT2D* (18%), *NOTCH1* (16%), *LRP1B* (14%), and *FAT1* (14%), similar to HNSCC top 5 genes. The most frequently identified *TP53* somatic mutations are R175H, R282W, Y220C, R213^*^, R248Q, R248W, R273H, and *R*342^*^ (51). Of these, *R175H, R248Q, R248W*, and *R273H* somatic alterations are also the most frequently encountered *TP53* alterations in HNSCC (18, 67). A recent systematic review has presented the clinical utility of circulating cfDNA as a novel biomarker for esophageal cancer (67). In total, 59 abstracts and 20 full papers have been reviewed including ESCC and esophageal adenocarcinoma cases. Seven studies have included patients with ESCC.

The most significant studies reporting the utility of cfDNA in ESCC are depicted in Table 3. In a cohort of 137 patients with ESCC, Tian et al. have shown that the use of 5-hydroxymethylcytosine (5hmc)-based biomarkers has clinical utility for diagnosis of ESCC, achieving a sensitivity of 93.75% and a specificity of 85.71% (Area Under Curve = 0.972). It has been shown that 5hmC, the oxidative product of 5-methylcytosine (5 mC), reflects the epigenetic features in patients with cancer, as it is a relatively stable intermediate of active DNA demethylation, being currently regarded as a novel epigenetic hallmark of cancer (73).

**Table 3:** Significant studies on cell-free DNA in ESCC—clinical applications.

**Nr**	**Authors**	**Year**	**Type of alteration**	**Type of cancer**	**Genes**	**Stage of disease**	**Assay type**	**Timing of sampling**	**Utility**
2	Hagi et al. (68)	2019	Mutation	ESCC	*TP53*	-	NGS with molecular barcodes	Before surgery	Diagnosis
3	Tian et al. (69)	2018	Methylation	ESCC	*5hmC*	-	Nano hmC Seal	Before treatment	Diagnosis
1	Li et al. (70)	2014	Methylation	ESCC	*EPB41L3, GPX3, COL14A1*	I-II	Infinium Methylation 450K array	Before treatment	Diagnosis
5	Liu et al. (71)	2011	Methylation	ESCC	*SFRP1; WIF1; DKK3, RUNX3*	I-IV	Methylation-specific PCR	Before treatment	Prognosis
4	Hibi et al. (72)	2001	Methylation	ESCC	*p16*	-	Methylation-specific PCR	Before surgery	Diagnosis and monitoring

In a pilot cohort of 13 patients with ESCC patients prior of resection and neoadjuvant therapy, Ueda et al. have shown that NGS using a panel of 53 cancer-related genes is an effective tool for detecting somatic alterations in plasma cfDNA. The assay was able to detect variant frequencies of 0.12–7.2% and the use of cfDNA analysis in clinical assessments of the tumor burden could help predict tumor recurrence in ESCC (74). The best diagnostic utility was identified for the presence of mutations in the following 4 genes *TP53, FAT3, MLL3*, and *AJUBA*. Using these somatic alterations, the authors achieved 78.9% sensitivity, 100% specificity, and 92.3% diagnostic accuracy. *TP53* is also a frequently mutated target in HNSCC, but *FAT1* and *FAT4* rather than *FAT3* are more frequently mutated in HNSCC, according to COSMIC.

In the study of Hagi et al., molecular barcode sequencing of the *TP53* gene enabled comprehensive and highly sensitive detection of ctDNA in patients with ESCC, prior to resection and neoadjuvant therapy as well as after therapy (68).

Luo et al. have designed a sequencing panel with improved targeting, identifying significantly mutated genes by meta-analysis of 532 ESCC genomes. The proposed gene panel consists of 90 genes, achieving 94 and 75% of sensitivity, respectively when detecting at least 1 or 2 mutant genes in patients with ESCC. Thus, the gene panel proposed by Luo et al. could be useful in ESCC diagnosis and disease monitoring following surgery (75). A comparison of pre- and postoperative plasma ctDNA using deep NGS sequencing has showed that some ctDNA mutations had a lower occurrence frequency or even disappeared postoperatively, providing useful cfDNA targets for disease recurrence.

Meng et al. have shown that cfDNA could potentially be used to monitor disease load in patients with ESCC, as somatic mutations could be detected in cfDNA of patients with stages IIA to IIIB presurgery, but at a lower frequency in cfDNA of the same patients postsurgery. Using an NGS panel of 483 genes they have shown that somatic alteration could be detected in cfDNA in genes involved in traditional cancer-related pathways: PI3K-Akt/mammalian target of rapamycin (mTOR) signaling, genes encoding proteoglycans or focal adhesion molecules, genes involved in FoxO signaling, chemokine signaling pathways, or Janus kinase - signal transducer and activator of transcription protein (JAK-STAT) signaling.

With the primary aim to determine whether detection of ctDNA after CRT is associated with risk of tumor progression Azad et al. have analyzed by NGS 802 hot spots in 607 genes for Single nucleotide polymorphisms (SNPs) previously associated with esophageal adenocarcinoma or squamous cell carcinoma, including hot spots in *LRP1B* and *TP53* frequently identified in HNSCC. The study group comprised of 10 ESCC cases out of 45 esophageal cancers. The authors have shown that the median proportion of tumor-derived DNA out of the total cell-free DNA before treatment was only 0.07%, further documenting the fact that ultrasensitive assays are needed for the assessment of ctDNA from localized esophageal tumors. The detection of ctDNA was associated with tumor progression, metastasis, and disease-specific survival (76).

Due to the lack of studies including patients with both conditions, currently, it is difficult to discriminate between HNSCC and ESCC based only on ctDNA mutation analysis.

## Noncoding RNA Expression in ESCC and HNSCC Liquid Biopsy

MicroRNAs are currently among the most promising biomarkers studied in many types of cancers, including ESCC and HNSCC, with diagnostic and prognostic values. The miRNAs are regulatory molecules found in tumoral cells and in the blood stream, carried by exosomes and function as translation repressors. MiRNAs can have multiple roles, being functional regulators of cell proliferation, interacting with many signaling pathways either as inhibitors or stimulators. Therefore, they can be upregulated or downregulated in various types of tumors (3, 77). MiRNAs can be quantified from formalin-fixed paraffin-embedded (FFPE) tumoral samples as well as from exosome or serum liquid biopsies, by qRT-PCR (78–80). Exosomes could be isolated using ultracentrifugation, filtration, chromatography, magnetic associated cell sorting, fluorescence, colorimetric ELISA assays, and other methods and are excellent miRNA carriers (3). Total exosomal RNA extraction followed by RT-PCR miRNA quantification is currently one of the best liquid biopsy approach for diagnosis and prognosis estimation in patients with cancer (81).

Our systematic literature search has identified 20 miRNAs and one lncRNA (*HOTAIR*) relevant for HNSCC and ESCC liquid biopsies. These miRNAs have multiple gene targets, in both ESCC and HNSCC.

*miR-10* is a promising biomarker for ESCC and HNSCC, targeting T-cell lymphoma invasion and metastasis-inducing protein1 (*TIAM1*). In ESCC *mir-10* down-regulates *TIAM1*, reducing its expression. Increased *TIAM1* expression is currently considered a negative prognostic factor in ESCC, as it is associated with increased histology grade, advanced clinical stages, and lymph node metastasis (82). Increased *miR-10* is a good prognostic indicator, being associated with the suppression of tumor growth and metastasis in ESCC via *TIAM1* repression. In HNSCC, *miR-10* was identified in patients with oral squamous cell carcinoma, having the same target as in ESCC, *TIAM1*, but also *HOXD10, RhoA/RhoC*, and *KLF4*. *miR-10* has also diagnostic utility as it achieved 81.2% sensitivity and 80% specificity in the diagnosis of ESCC and 94.4% sensitivity and 80% specificity for HNSCC detection (83, 84). The main miRNA targets with diagnostic utility in HNSCC and ESCC and proposed signaling pathways are presented in Table 4.

**Table 4:** MicroRNA and lncRNA liquid biopsy studies in ESCC/HNSCC—targets and signaling pathways, and diagnostic utility.

**Year**	**Type of RNA**	**Authors ESCC**	**Authors HNSCC/ type of HNSCC**	**Identified target gene ESCC**	**Identified target gene HNSCC**	**Signaling pathway ESCC/HNSCC**	**Diagnosis of ESCC**	**Diagnosis of HNSCC**
2021	*miR-122*	Wang et al. (85)	Ruales et al. (86); LSCC	*KIF22*	*Bcl-W, CCNG1*	EMT pathway PI3K/Akt/mTOR / p70S6K	–	–
2019	*miR-21*	Chen et al. (87); Wang et al. (88)	Avissar et al. (89)	*RASA1*	*PDCD4, PTEN, MARCKS;*	–	71% sensitivity and 96,9% specificity	–
2019	*miR-98*	Huang et al. (90)	Du et al. (91); OSCC	*EZH2 (High when mir98 is low)*	*IGF1R*	– PI3K-Akt, MAPK	–	–
2019	*miR-10*	Liu et al. (84); Zhang et al. (92)	Lu et al. (83); OSCC	*TIAM1*	*HOXD10, TIAM1, RhoA/RhoC, KLF4*	TIAM1-Rac1 –	81,2% sensitivity and 80% specificity	94,4% sensitivity and 80% specificity
2019	*miR-141*	Zhang et al. (93)	Ganci et al. (94); OSCC	*YAP1, SOX17*	*PTEN*	–	–	–
2019	*miR-139*	Jiao et al. (95)	Wang et al. (88); OSCC	*VEGFR1*	*HOXA9*	–	–	–
2019	*miR-375*	Luo et al. (96)	Wu et al. (97); OSCC	*ARL4C; SLC7A11*		Wnt/b-catenin and EGF/RAS signaling	–	–
2018	*miR-23*	Zhang et al. (98); miR23b	Zhang et al. miR23a (99); LSCC	*EBF3*	*APAF*	– –	–	–
2018	*miR-424*	Wen et al. (100)	Li et al. (101); LSCC	*PRKCD, WEE1*	*CADM1*	TGFb/SMAD activate miR-424 PI3K/Akt	–	–
2018	*miR-365a*	Sun et al. (102)	Geng et al. (103); LSCC	*PSAT1*	*p-AKT*	PI3K/Akt	–	–
2018	*miR-125b*	Fan et al. (104)	Arriagada et al. (105); OSCC, LSCC	*BMF (BCL2 modifying factor)*	*A20*	–; NF-kB	–	–
2018	*miR-196a*	Wang et al. (106)	Hu et al. (107)	*lncRNA GAS5*	*ANXA1*	–	–	–
2018	*miR-34a*	Zuo et al. (108)	Kalfert et al. (109)	*CD44*		NF-kB; –	–	–
2018	*miR-455*	Liu et al. (110)	Cheng et al. (110); OSCC	*Rab31; CGHNK2; OEC-M1; SCC15; TW2.6*		–; TGFB/SMAD pathway	–	
2017	*miR-9*	Cui et al. (111)	Sun et al. (112); OSCC	*E-cadherin*	*CXC chemokine receptor 4*	NF-kB, MAPK14, PI3K, JAK STAT, Hippo;; Wnt/B-cathenin	85.5% sensitivity and 98.5% specificity	–
2017	*miR-200c*	Zheng et al. (113)	Lo et al. (114)	*P21*	*BMI1*	G2/M checkpoint pathaway; ZEB1, ZEB2 pathways in EMT signaling	–	–
2017	*miR-99b*	Ma et al. (115); miR99b/let-7e/mir-125a cluster	Jakob et al. (116); OSCC	*ARID3A, ZEB1*		–; Glycogen synthase kinase 3b	–	–
2017	*HOTAIR (lncRNA)*	Wang et al. (117)	Wang et al. (118); LSCC	*HOTAIR*		– –	Sensitivity 56%, specificity 90%	Sensitivity 94,2%; Specificity 73,5%
2016	*miR-7*	Dong et al. (119)	Jiang et al. (120)	*EGFR*	*IGF1R*	KLF4 and NF-kB; EGFR cell signaling	78,1% sensitivity and 83,3% specificity	–
2016	*miR-146a*	Liu et al. (82)	Ruales et al. (86)	*IRS2; IL1*	*Kinase-1 associated with IL1 receptor*	EGFR and Wnt/b-cathenin; NFkB pathaway; NFkB pathaway	–	–
2016	*miR-574*	Okumura et al. (78)	Summerer et al. (121)	–		Wnt signaling pathway/EGFR –	93% sensitivity and 80% specificity	–

A significant signaling pathway for ESCC and HNSCC pathogenesis is controlled by the transcription factor NF-kB and was found to be regulated by *miR-7, miR-9, miR-146a*, and *miR-34a* in ESCC and by *miR-125b* and *miR146a* in HNSCC, respectively. Thus, *miR-146a* is involved in both malignancies via NF-kB pathway regulation, but it seems to have an opposite behavior, as it is downregulated in ESCC and up-regulated in HNSCC (82, 86, 105, 108, 111, 119).

Furthermore, among the evaluated miRNA targets, *miR-9, miR-21, miR-23, miR-141, miR*-*424*, and *miR-196a* were upregulated in both the cancers, whereas *miR-7, miR-98, miR-122, miR-139, mir-200c*, and *miR375* were downregulated in both the ESCC and HNSCC. These miRNA targets were associated with metastasis, invasion, tumoral cell growth, and poor prognosis in both the tumor types (85–87, 89, 90, 93–95, 98–101, 106, 107, 111–114, 122). Other miRNA targets could have utility for diagnosis or prognostic stratification as they were up-regulated in HNSCC and down-regulated in ESCC: *miR-10, miR-365a, miR-125b, miR-146a, miR-34a, miR-455*, and *miR574*. On the other hand, *miR-99b* was upregulated in ESCC and downregulated in HNSCC (78, 82–84, 86, 102–105, 108–110, 121, 123).

The potential roles of miRNAs in diagnosis, prognosis, and as possible therapeutic targets are given in Table 5. Of particular interest as a possible target for treatment is *miR-200c* since it was associated in both types of SCC with increased radiosensitivity. It controls G2/M checkpoint in DNA replication via p21 and BMI1 as well as ZEB1 and ZEB2 signaling pathways. *miR-200c* is downregulated in aggressive SCCs and its increased expression is associated with a better prognosis and a better response to radiotherapy (113, 114).

**Table 5:** MicroRNA and long non-coding RNA (lncRNA) liquid biopsy studies in ESCC/HNSCC—outcomes and clinical utility.

**Year**	**Type of RNA**	**Authors ESCC**	**Authors HNSCC/type of HNSCC**	**Outcome ESCC**	**Outcome HNSCC**	**Utility in ESCC**	**Utility in HNSCC**
2021	*miR-122*	Wang et al. (85)	Ruales et al. (86); LSCC	Down-regulation of *KIF22* restrained malignant progression	Inhibition of tumorigenesis; role in diagnosis	Prognosis and treatment target	Diagnosis
2019	*miR-21*	Chen et al. (87)	Avissar et al. (89)	Role in proliferation, migration, invasion; Possible target of therapy	High expression - poor prognostic; Higher expression in OSCC	Diagnosis, Prognosis and treatment	Prognosis
2019	*miR-98*	Huang et al. (90)	Du et al. (91); OSCC	Tumor suppressor gene; Overexpression inhibit migration and invasion	Suppress cell migration and invasion	Prognosis	Prognosis
2019	*miR-10*	Liu et al. (84); Zhang et al. (45)	Lu et al. (83); OSCC	Overexpression suppresses cell proliferation and enhances apoptosis; Potential target of therapy	Increase cell migration and invasion;	Diagnosis, Prognosis and treatment	Diagnosis, Prognosis
2019	*miR-141*	Zhang et al. (93)	Ganci et al. (94); OSCC	Promotes cell proliferation and inhibits apoptosis Potential target of treatment	High expression correlates with shorter survival; Predict local recurrence	Target of treatment	Prognosis
2019	*miR-139*	Jiao et al. (95)	Wang et al. (122); OSCC	Role in inhibition of ESCC; Therapeutic target	Inhibits tumorigenesis and progression	Target of treatment and prognosis	Target of treatment
2019	*miR-375*	Luo et al. (96)	Wu et al. (97); OSCC	Low expression correlates with poor prognosis Acts as tumor suppressor	Downregulated in OSCC cells - tumor suppressor	Prognosis	Diagnosis and prognosis
2018	*miR-23*	Zhang et al. (98); miR23b	Zhang et al.; miR23a (99); LSCC	Upregulation in ESCC and can induce proliferation, invasion and metastasis	Poor prognosis, greater extent of lymph node, worse clinical stage, short survival	Proliferation and metastasis	Prognosis
2018	*miR-424*	Wen et al. (100)	Li et al. (101); LSCC	Upregulation correlates with poor survival	High level–poor differentiation, advanced tumor stage	Prognosis and target of treatment	Target of treatment
2018	*miR-365a*	Sun et al. (102)	Geng et al. (103); LSCC	High levels inhibited cell invasion, colony formation and growth of tumoral cells	Promoted tumor growth and metastasis	Biomarker of development and progression	Target of treatment
2018	*miR-125b*	Fan et al. (104)	Arriagada et al. (105); OSCC, LSCC	Expression decreased; Overexpression inhibited tumor growth	High level–poor prognosis; metastasis, lower survival	Target of treatment and prognosis	Progression and metastasis
2018	*miR-196a*	Wang et al. (106)	Hu et al. (107)	Suppress expression of *GAS5* which functions as tumor suppressor gene. Is upregulated in ESCC tumor cells	Downregulation suppressed proliferation, invasion and migration of EC109 cells. May function as oncogene;	Diagnosis and target of treatment	Target of treatment
2018	*miR-34a*	Zuo et al. (108)	Kalfert et al. (109)	Inhibited cell invasion, migration, tumor growth and metastasis, by regulating CD44	Higher levels in p16 positive OSCC type. Acts as tumor suppressor	Prognosis	Prognosis and diagnosis
2018	*miR-455*	Liu et al. (110)	Cheng et al. (110); OSCC	Overexpression inhibited proliferation, migration and invasion of Eca109 cells	Enhances proliferation and cell growth	Diagnosis and prognosis	Diagnosis
2017	*miR-9*	Cui et al. (111)	Sun et al. (112); OSCC	High value–poor tumor differentiation, large size, deep invasion, lymph node, poor prognosis	low value- poor prognosis; tumor suppressive role	Diagnosis and prognosis	Prognosis
2017	*miR-200c*	Zheng et al. (113)	Lo et al. (114)	Enhances radiosensitivity; Downregulation associated with radiotolerance; Effects in tumor migration and invasion inhibition	Overexpression reduces tumourigenicity and metastasis; enhances radiosensitivity; Role in radio-chemo-resistance	Target of treatment	Target of treatment
2017	*miR-99b*	Ma et al. (115); miR99b/let-7e/mir-125a cluster	Jakob et al. (116); OSCC	Role in tumor metastasis. Enhanced cell mobility	Downregulation is associated with increased proliferation and colony formation	Prognosis	Prognosis
2017	*HOTAIR; (lncRNA)*	Wang et al. (117)	Wang et al. (118); LSCC	Potential biomarker of diagnosis;; Correlated with TNM stage;; Decreased after surgery	Higher levels in advanced stages; Correlates with miR-21	Diagnosis	Diagnosis and prognosis
2016	*miR-7*	Dong et al. (119)	Jiang et al. (120)	lower level correlates with longer tumor and status of lymph node	lower level correlates with increased risk, faster progression; independent of localization, T stage, treatment	Diagnosis and prognosis	Prognosis
2016	*miR-146a*	Liu et al. (82)	Ruales et al. (86)	Suppressed EC109 and TE8 cell proliferation and invasion; Inhibited the expression of *IRS2*	Overexpressio–progression to metastasis	Target of treatment and diagnosis	Diagnosis and prognosis
2016	*miR-574*	Okumura et al. (78)	Summerer et al. (121)	Tumor suppressor effect;	Reduced survival and poor prognosis after treatment; HPV independent marker	Prognosis	Prognostic

Homebox - HOX transcript antisense RNA (HOTAIR), a lncRNA was identified as a valuable biomarker for both HNSCC and ESCC, having regulatory effects in tumoral cells, by controlling proliferation, invasion, and differentiation. *HOTAIR* expression was directly correlated to the TNM stage. Its serum expression in patients at risk could be used also as a diagnostic tool, with a sensitivity of 56 and 94.2% and specificity of 90 and 73.5% for ESCC and for HNSCC, respectively (15, 117, 118).

In the future, circulating non-coding RNA expression could be one of the best methods to discriminate between ESCC and HNSCC and robust research studies including patients with both conditions and cohorts in which the two malignancies occur synchronous or metachronous are mandatory.

## Conclusion and Future Research Direction

Liquid biopsy as a field of research could be an important tool in the battle of discovering new biomarkers for early diagnosis, since both the HNSCC and ESCC are currently diagnosed in a late stage of disease, with advanced tumor locoregional invasion, associated with complications, metastases, and poor survival. Currently, in the approved clinical guidelines, there are no biomarkers proposed for early diagnosis in patients that are at risk. There are excellent reviews focused on liquid biopsy applications for individual malignancies. Recently, Mishra et al. have reviewed clinical applications of different liquid biopsy multifunctional biomarkers in head and neck cancers such as: somatic DNA mutations-based markers, methylation profiles, mitochondrial DNA-based biomarkers, exosomes and EVs, transcriptomic, nongenomic based biomarkers as well as CTCs, and viral-based biomarkers (124). Our extensive literature search, however, has identified no study, so far, addressing the topic of common or specific molecular biomarkers validated in patient cohorts with synchronous or metachronous HNSCC and ESCC, or at least studies including patients with both malignancies. This is still an unmet need, although one could imagine significant clinical applications in terms of early diagnosis and prognostic stratification. The present review is the first that has aimed to analyze current knowledge regarding the utility of liquid biopsy in both conditions and to provide potential targets for further research. The review was focused on common markers, as these could be identified based on published literature, in the lack of studies systematically addressing both malignancies with the same panel of molecular markers.

The current applications, strengths, and limitations of liquid biopsies have been recently reviewed by Martins et al. (125). The main strength of liquid biopsies resides in the non-invasive character of the procedures that could be applied repeatedly to capture the molecular dynamics of the tumors. This is a major point with clinical impact considering the changing molecular characteristics of the tumors subjected to multimodal therapies. In comparison to traditional biopsies, liquid biopsies have the potential to better capture the intratumor molecular variability at different time points during follow-up. However, liquid biopsies has not yet fully entered daily practice as they require expensive equipment and trained personnel to conduct tests and interpret the results. There are also technology-specific limitations. CTCs are rarely found in the circulation and their isolation is difficult and costly. For cfDNA pre-analytical protocols lack standardization whereas the cfDNA template is found in specific body fluids at very low concentrations, leading to the low sensitivity of detection. Techniques to identify and isolate RNA species are still under development and one of the main challenges is the instability of the RNA molecule leading to difficulties in the identification of low abundance RNA species. The isolation and characterization of extracellular vesicles show great promise in the field of liquid biopsy. EVs could derive specifically from the tumor cells and are excellent carriers of tumor-related molecular information, but the lack of standardization of preanalytical protocols is still challenging.

The detection and quantification of CTCs have a minor role as a diagnostic tool, since they are usually discovered in the late stages of the disease and are related to extensive tumor spread and metastasis, having mainly a prognostic utility. The use of the FDA-approved Cellsearch platform has a good applicability for both ESCC and HNSCC.

Circulating tumor DNA analysis shows promising perspectives for early diagnosis as studies have already indicated both common and specific somatic alterations but identified somatic variants should be further validated in independent series comprising both malignancies.

The investigation of methylation profiles seems to have the best utility for early diagnosis of both pathological conditions. Two years ago, using a ctDNA plasma methylation platform, the feasibility of cancer detection up to four years before the clinical cancer diagnostic in five common malignancies including esophageal cancer has been demonstrated (126). Almost at the same time, the results of an article that is a potential game changer have been published in the Annals of Oncology (127). The study included 6689 participants (2482 with more than 50 types of cancer and 4207 without cancer. The study used methylation patterns in cfDNA. The specificity of cancer detection was close to 100% and the sensitivity was 67.3% in 12 types of cancer including ESCC and HNSCC. The study did not specify whether any patients with both ESCC and HNSCC were included. Aside from methylation, TP53 somatic alterations could be proposed for screening purposes along with other somatic variants in frequently mutated genes such as *KMT2D, NOTCH1, LRP1B*, and *FAT1*. Specific polymorphisms could be integrated in screening panels with applicability for early diagnosis, detection of residual disease following surgery, or treatment response after radio-chemotherapy.

Given the relationship of some head and neck cancers with viral infections, (Epstein-Barr virus (EBV) for nasopharynx cancers and human papilloma virus (HPV) for SCC of the oropharynx), it is likely that in the near future biomarkers related to these infections will be used routinely in the diagnostic and management of viral related cancers. Five years ago, a landmark article published in New England Journal of Medicine demonstrated the feasibility and usefulness of screening plasma EBV ctDNA in a population at high risk for nasopharynx cancer (128). Although less than 1/1000 HPV carriers develop cancer, at least in theory, a similar technique may be used also for early detection of HPV ctDNA in a population at risk, for example HPV carrier men (129).

Possibly the best field of research is translational regulation by noncoding RNAs, with applications for early diagnosis and prognostic stratification of both the patients with HNSCC and ESCC. There are currently multiple miRNA targets identified that behave similarly in both cancers as well as targets with opposite effects, some of them showing promising utility even as therapeutic targets.

However, none of the biomarkers described in this review can be used individually to robustly discriminate between ESCC and HNSCC. Further research is needed to integrate currently available molecular targets in biomarker panels, with clinical applicability for screening purposes and early diagnosis, detection of residual disease, and prognostic estimation. Given the different prognostic of these conditions, the discovery of specific molecular markers for each of these two malignancies remains an unmet need of paramount importance.

## Author Contributions

RI and MM have conducted the systematic literature search and generated the first draft of the manuscript. SI has updated CTC section of the manuscript and provided clinical background for the ESCC sections. CP has updated the cfDNA section. RH has conceived the general plan of the article, updated the miRNA section, and provided clinical background for the HNSCC sections. DP and CG have supervised the study and provided critical comments and review of the manuscript. All authors edited the manuscript and approved the submitted version.

## Conflict of Interest

The authors declare that the research was conducted in the absence of any commercial or financial relationships that could be construed as a potential conflict of interest.

## Publisher's Note

All claims expressed in this article are solely those of the authors and do not necessarily represent those of their affiliated organizations, or those of the publisher, the editors and the reviewers. Any product that may be evaluated in this article, or claim that may be made by its manufacturer, is not guaranteed or endorsed by the publisher.
